# Effects of Bromelia pinguin (Bromeliaceae) on soil ecosystem function and fungal diversity in the lowland forests of Costa Rica

**DOI:** 10.1186/1472-6785-14-12

**Published:** 2014-05-05

**Authors:** Caitlin I Looby, William D Eaton

**Affiliations:** 1 Department of Ecology and Evolutionary Biology, University of California-Irvine, Irvine, CA 92697, USA; 2 Biology Department, Pace University, New York, NY 10038, USA

**Keywords:** Carbon biomass, Nitrogen biomass, *Bromelia pinguin*, Fungal diversity, Soil ecosystems

## Abstract

**Background:**

*Bromelia pinguin* (Bromeliaceae) is a terrestrial bromeliad commonly found under forest stands throughout the Neotropics that has been shown to have antifungal activity in vitro. We have hypothesized that this bromeliad would also have an effect on the fungal populations in nearby soil by decreasing fungaldiversity and negatively impacting C and N cycle-related activities. A previous study in the lowland forest of Costa Rica showed the soil beneath these bromeliads had decreased fungal ITS DNA and differences in C and N levels compared to adjacent primary forest soils.

**Results:**

In this follow-up study, we found that the bromeliad soils had lower rates of C and N biomass development and lower phenol oxidase activity (suggesting less decreased fungal decomposition activity). The results of T-RFLP and cloning-based taxonomic analyses showed the community level diversity and abundance of fungal ITS DNA was less in bromeliad soils. Sequence analysis of fungal ITS DNA clones showed marked differences in fungal community structure between habitats of Basidiomycota (Tremellales, Agricales, Thelephorales), Ascomycota (Helotiales), and Zycomycota populations.

**Conclusions:**

The data show there to be differences in the soil nutrient dynamics and fungal community structure and activity associated with these bromeliads, as compared to the adjacent primary forest. This suggests the possibility that the anti-fungal activity of the bromeliad extends into the soil. The bromeliad-dense regions of these primary forest habitats provide a unique natural micro-habitat within the forests and the opportunity to better identify the role of fungal communities in the C and N cycles in tropical soils.

## Background

Biodiversity is unequally distributed across latitudes, with the tropics consisting of the most biologically diverse ecosystems on earth [1,2]. Vascular plants are a fundamental component of the high biodiversity and species richness present in tropical regions, which have been described as regions where “common species are rare and rare species are common” [2,3]. This high level of aboveground biomass, diversity, and net primary production [4] is inextricably linked and allows for more efficient biogeochemical cycling [5]. Tropical ecosystems contribute more to global nutrient cycling and storage in comparison to other ecosystems [6]. It has been estimated that up to 30 percent of the world’s carbon (C) is stored within the top 200 cm of all tropical soils [7,8]. Despite the importance of these tropical ecosystems, it is still unclear how local differences in aboveground communities, or anthropogenic disturbances to them, can impact the functioning of the nutrient cycle systems [1,9]. Gaining a clearer understanding of how local changes in aboveground communities impact soil biota and nutrient processes would enhance our understanding of nutrient cycle dynamics and the drivers of these systems.

The great diversity and complex interactions assumed to occur between above- and belowground communities [10,11] especially within in tropical ecosystems, make it difficult to evaluate how communities affect each other and ecosystem processes. It is thought that changes in plant species composition and diversity can impact the belowground community and important ecosystem processes such as decomposition [10,12].

Some studies have shown how plant species alter the structure of belowground communities and ecosystem function [13-16]. In particular, it has been demonstrated how changes in agroecosystem and grassland plant assemblages and the invasion of new species can reduce microbial community diversity and ecosystem function [17-24]. Most work has been done in these disturbed systems rather than on naturally occurring plant species within undisturbed forests to determine if naturally occurring plants can have such influences on microbial communities and ecosystem functions.

Bromeliaceae is a Family of plants important to the diversity of vascular plants in the tropics [25]. This family consists largely of epiphytic plants and has shown explosive radiation throughout the Neotropics, with most of the approximately 2700 species being endemic to this region [25,26]. Although a majority of these species are epiphytes, there are several species within this family that are terrestrial; the most well known being the pineapple plant, *Ananas comosus*. Another terrestrial representative of this Family is *Bromelia pinguin*, a terrestrial bromeliad found under forest stands throughout Central America and the Caribbean. Known as maya or piñuela, *B. pinguin* has many unique characteristics including a very acidic fruit and spiny fronds that extend from the center of its tank-like structure [27]. Due to their large size and sharp fronds, these bromeliads were used as hedges by the Maya to protect their land from large mammals, and more recently the fruit has been used to make drinks [27]. Although this plant has had practical and beneficial uses for humans, the ecological role of this bromeliad is unclear.

It was discovered in an *in vitro* assay that the fruit pulp of *B. pinguin* had antifungal properties [28]. A previous study conducted by Looby *et al.*[29] suggested that these antifungal properties might also be exhibited in the soil. In a comparison between soil underneath bromeliad-dense patches and an adjacent primary forest it was determined through qPCR-based analyses that bromeliad soil had decreased amounts of fungal DNA (using universal fungal 18 s rRNA and fungal ITS primers), increased soil dissolved organic C (DOC), decreased standing C biomass (Cmic), and decreased efficiency of C use (higher qCO_2_ and lower Cmic to DOC ratio). These results suggested the difference in fungal community structure might be associated with greater utilization of more labile forms of organic material in the bromeliad soils, and a reduced capacity to decompose more complex organic matter.

In this current study, soil was collected at increasing distances from dense bromeliad patches, within a primary forest of the Maquenque National Wildlife Refuge (MNWLR) within the San Juan-La Selva Biological Corridor (SJLSBC) in the Northern Zone of Costa Rica, to evaluate the impact *B. pinguin* has on fungal community structure and rates of C and N utilization. We hypothesized that these bromeliad-associated soils would have lower rates of C and N biomass development and less diversity and richness of fungi. Extracellular phenol oxidase activity has been associated with wood-rotting fungi for some time [30]. Thus, we also hypothesized there to be less phenol oxidase activity associated with the bromeliad soils, as a result of the potential anti-fungal activity of the plants.

## Methods

### Study sites and sampling

**Figure 1:**
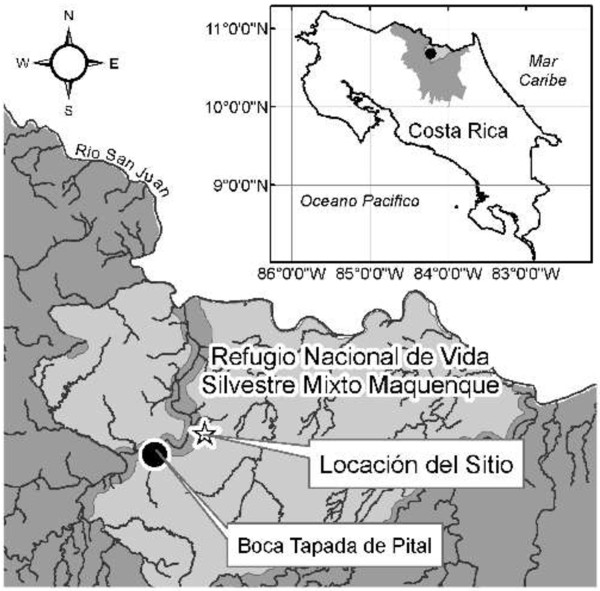
**Location of field sites (Locación del Sitio) in the Maquenque National Wildlife Refuge (Refugio Nacional de Vida Silvestre Mixto Maquenque) in Costa Rica.** Map courtesy of the Dr. Olivier Chassot, Centro Cientifico Tropical, and the San Juan-La Selva Biological Corridor Commission.

As a result of four decades of extraction-based land practices in the region, the SJLSBC was established to protect Northern Zone ecosystems, promote sustainable practices while still allowing land to be privately owned, and to provide a continuous stretch of forest between Costa Rica and Nicaragua. This study was conducted in an old-growth lowland forest that has not been harvested within the MNWLR (Figure 1; approximately N 10° 41' 11'', W 84° 12' 22'', 15 km south of the Nicaraguan border), an area within the SJLSBC that has been identified as one of the corridor’s most valuable areas for biodiversity due to it containing the most intact forest within the SJLSBC [31].

Fieldwork was conducted in July 2010, in four distinctly separated primary forest regions, each containing sections with dense patches of *B. pinguin* adjacent to areas lacking this plant. Four 20 m transects were established in each of these four regions: one along the center of a bromeliad dense region, one along the edge of a bromeliad dense patch, one 3 m away from the edge, and one 7 m away from the edge. For this study, the edge was defined as a transect that contained less than 10 bromeliads. Densities of *B. pinguin* were determined along each transect; bromeliads were counted if their base was present within 1 m of the transect. Detailed information regarding bromeliad abundance is shown in Table 1. Twenty 2 cm wide × 15 cm deep soil cores were collected along each 20 m transect to include the O horizon and the top 5 to 10 cm of the A layer. The 20 cores from each transect were composited and homogenized prior to analysis. Composited samples were sieved at 5 mm prior to nutrient analysis and DNA extraction. All measured nutrient values were adjusted for dry weight. Percent soil moisture and pH were measured using a Kelway HB-2 soil and pH meter (Wyckoff, NJ, USA).

**Table 1:** **Densities of *Bromelia pinguin *** **along transects, to assess its impact on nutrient activities rates**

**Plot**	**Center of bromeliad- dense transect**	**Edge of bromeliad- dense transect**	**3 m transect**	**7 m transect**
A	20	17	5	0
B	21	11	1	0
C	20	16	7	4
D	22	18	8	2

Soils were grouped into “bromeliad” and “primary forest” soils for all analyses. The “bromeliad” soils consisted of the center and edge samples (which were composited), and the “primary forest” soils consisted of the samples collected 3 and 7 m from the bromeliad edge (which were composited). This approach allowed us to ensure samples were included from across the bromeliad patches, and not just focus on high density bromeliad sections.

### Rates of C and N biomass development and phenol oxidase activity

The amount of microbial biomass N (Nmic) was determined by the potassium thiosulfate oxidation methods of Jiménez *et al*. [32], measuring the difference in Total N in chloroform-fumigated and unfumigated samples. Measurements were conducted using the ammonium salicylate and cadmium reduction spectrophotometric methods from the HACH DR 2700 system (HACH Company, Loveland, CO, 80539-0389; HACH method 8192). To determine the rate of Nmic development, soil was incubated for 9 days and the differences in Nmic from unincubated and incubated samples were determined.

The amount of microbial biomass C (Cmic) was measured as the difference between soil DOC in chloroform fumigated and unfumigated samples. The Walkley-Black rapid dichromate procedure, modified by Nelson and Sommers [33], was used to measure levels of DOC within the samples. Rates of Cmic development were determined as the difference in Cmic in soil that was unincubated and incubated over 9 days. Phenol oxidase activity was measured over time, using the methods of Saiya-Cork *et al.*[34] and Sinsabaugh [35], to suggest differences in the level of fungal-associated degradation of woody materials. Three wavelengths were used to determine this activity (415, 436, and 600 nm) and the average change in absorbance was calculated.

### Fungal ITS community level and taxonomic diversity analyses

DNA was extracted from the composited soil samples using the Power Soil DNA Isolation kit (MO BIO, Carlsbad, CA) and was stored at 4°C prior to downstream analysis. Concentration and purity (A_260_/A_280_ ratio) of the extracted DNA were determined using a Nanodrop 2000/2000c (Thermo Scientific, Waltham, MA). DNA was pooled into two groups, center and edge transects and 3 m and 7 m transects, to measure differences in community level and taxonomic level diversity of fungal ITS target DNA in these two habitat types.

Fungal community diversity was evaluated by polymerase chain reaction (PCR) targeting fungal ITS target DNA followed by terminal restriction fragment length polymorphism (T-RFLP) and Sanger sequencing of clones. For both analyses, the PCR we used the ITS1f and ITS4 primer set to amplify ITS region of the rRNA in fungi [36], and the specific amplification conditions by Gardes and Bruns [36] and Looby *et al*. [29]. For T-RFLP, the ITS1f primer was fluorescently labeled with 6-FAM (Applied Biosystems, Inc., Foster City, CA). FAM-labeled PCR product was purified using the Ultra PCR Clean-Up Kit (Thermo Scientific, Waltham, MA) prior to restriction digestion.

Fluorescently labeled PCR products were digested using Hinfl, Alul, and Taq restriction enzymes (Fermentas) and the manufacturer’s instructions. Digested FAM-labeled PCR product was diluted 1:10 μL and then 1 μL of diluted PCR product was mixed with 1 μL GeneScan-500 LIZ size standard and Hi-Di Formamide to a final volume of 10 μL. Samples were heat denatured for 3 min at 95°C prior to analysis. Data were collected on a 3130 Genetic Analyzer (Applied Biosystems, Foster City, CA) and fragment profiles were analyzed using GeneMapper Software 4.1 (Applied Biosystems, Foster City, CA).

In a preliminary cloning experiment, we used the pGEM-T Easy Vector System (Promega, Madison, WI) to develop PCR insert DNA clone libraries. The DNA sequences were processed and identified using the BigDye 3.1 system and a 3130 Genetic Analyzer (Applied Biosystems, Foster City, CA). Sequences were aligned using Laser Gene software (DNASTAR Inc, Madison, WI) and were subjected to BLAST analysis within the National Center for Biotechnology Information (NCBI) database for phylogenetic inferences and grouped by order as Operational Taxonomic Units (OTU). Only 40 clones were sequenced per habitat in this preliminary study, to obtain the first snapshot estimate of the fungal Orders present in these two habitat types. We recognize that the number of clones examined in this study represents a small component of the fungal population within these soil communities, but made the assumption that due to the increased chance of being cloned, they represented the most common species within these soils.

### Data analysis

Based on the methods of analysis for small data sets suggested by Di Stefano *et al.*[37], estimations of the biological importance of the mean differences in the various metrics was determined using Students’ *t*-tests *p* value, percent difference (PD), and effect size (Hedges’ *g*) as determined in SPSS 16.0 (IBM Corporation, New York, United States). This approach is used on small data sets as the traditional “statistical significance” methods may result in *p* values > 0.05 to 0.1. In this approach, the combination of *p* and *g* values are most critical as the *p* value describes the probability that an effect is present and the *g* value the size of the effect. For this study, we defined “important biological differences” as differences between mean values that had *p* < 0.15, PD > 40%, and *g* > 0.75 (as >0.7 is considered a large effect size difference). Given the small sample sizes associated with this study, we wanted to ensure that we were being conservative in our estimates of the importance of the mean differences of the biogeochemical metrics used. Thus, *post-hoc* Power Analyses were conducted to determine the probability of committing Type II errors (false negatives). A Pearson’s correlation analysis was conducted as a simple method to determine if bromeliad densities were associated with the differences in the biogeochemical parameters measured. Standard measurements of diversity (Shannon Diversity), richness (ACE), and dominance (Simpson Dominance) were used to estimate community level and taxonomic diversity of fungal ITSusing EstimateS [38].

## Results

### Soil properties and bromeliad densities

Soil moisture and pH were not different between transects and habitat type according to the weight of evidence used to determine important, biological differences (data not shown). Densities of *B. pinguin*, as expected were far less in the primary forest transects than in the transects associated with the bromeliad patches (Table 1).

### Rates of biomass development and phenol oxidase activity

Rates of Cmic and Nmic development were compared by habitat type (bromeliad dense transects and primary forest transects). The rates of Cmic and Nmic development were lower in the bromeliad soils (Table 2), which had a 47.9% lower rate of Cmic and 41.8% lower rate of Nmic development than primary forest soils. There was 80.7% less phenol oxidase activity in the bromeliad soils (Table 2). Correlation analysis showed that increased bromeliad density was negatively correlated with rates of Cmic and Nmic development and phenol oxidase activity (Table 2). The statistical Power Analyses levels for these comparisons were 0.62, 0.72, and 0.64, respectively.

**Table 2:** Mean rates of Cmic and Nmic development and laccase activity in bromeliad-dense and primary forest soils

**Parameters measured**	**Bromeliad (Mean ± SD)**	**Primary (Mean ± SD)**	**PD (%)**	**Students’ t-test ( *p *)**	**Hedges’ effect size ( *g *)**	**Pearson’s correlation with bromeliad density ( *r *, *p*)**
Cmic development	60.70 ± 11.50 μg/g/day	89.79 ± 49.42 μg/g/day	47.9	0.13	0.77	-0.375, 0.15
Nmic development	20.54 ± 11.01 μg/g/day	29.04 ± 8.61 μg/g/day	41.8	0.10	0.81	-0.479, 0.06
Laccase activity	0.00238 ± 0.00134 ∆OD/min	0.00429 ± 0.00238 ΔOOD/min	80.7	0.07	0.82	-0.457, 0.07

### Fungal ITS community level and taxonomic diversity

There were clear differences in the fungal community composition and structure between bromeliad and primary forest soils as determined by T-RFLP (Table 3). Bromeliad soils had decreased fungal ITS diversity, richness, and evenness of distribution of fungal groups in comparison to primary forest soils (Table 4). This pattern was consistent with the cloning analysis results (Table 4).

**Table 3:** Terminal restriction fragment length polymorphism analyses of fungal DNA in bromeliad and primary forest soils

**Bromeliad**		**Primary**	
**Fragment length (bp)**	**Area of fluorescence**	**Fragment length (bp)**	**Area of fluorescence**
41.16	1374	41.17	436
172.05	625	61.89	314
185.82	827	168.89	959
248.29	508	172.01	379
296.66	1928	181.48	396
299.06	1427	185.77	2124
		201.54	542
		203.57	396
		248.19	1340
		255.93	513
		315.25	702
		369.09	3290

**Table 4:** T-RFLP and DNA sequence-based richness, diversity and dominance indices from bromeliad and primary forest soils (±SD)

		**T-RFLP**		**Sequencing**	
		**Bromeliad**	**Primary**	**Bromeliad**	**Primary**
Richness					
	ACE	7.40 ± 0.68	12.00 ± 1.8	229.2 ± 17.7	396.6 ± 29.2
Diversity					
	Shannon Diversity (ln) Index	1.73 ± 0.23	2.24 ± 0.16	3.07 ± 0.34	3.77 ± 0.26
Dominance					
	Simpson Dominance Index	0.63 ± 0.04	0.84 ± 0.06	0.97 ± 0.04	0.99 ± 0.03

The differences in the relative abundances of fungal orders, suggest a shift in the fungal community structure between the bromeliad and primary forest soils. Fungal groups from the phylum Basidiomycota dominated the bromeliad soil clones, while fungi from the phylum Ascomycota dominated the primary forest soil clones (Table 5). The composition of the different fungal orders were also different in each soil type. The most common Basidiomycete orders found in bromeliad soils were Tremellales and Agaricales and the most common order in the phylum Ascomycota found in bromeliad soils was Hypocreales. There were no representatives from any of these three orders found in primary forest soil clones. As well, clones representing the phyla Zygomycota, Glomermycota, and Chytridiomycota were only found in the bromeliad soils. In the primary forest soil clones, Helotiales (phylum Ascomycota) and Thelephorales (phylum Basidiomycota) were the most common orders represented. Only 5% clones from the bromeliad soils were from the order Thelephorlaes, and none were from the order Helotiales.

**Table 5:** Relative abundance of fungal DNA by phyla and order in bromeliad and primary forest soils

**Classification**		**Habitat type**			
**Phylum**	**Order**	**Bromeliad**		**Primary**	
		**%**	**OTU**	**%**	**OTU**
Basidiomycota	Agricales	22.5	5	5.6	
	Boletales	0	0	2.8	1
	Corticales	2.5	1	0	1
	Class: Exobasidiomycetes	0	0	5.6	0
	Thelephorales	5.0	2	25	2
	Trechesporales	5.0	2	0	8
	Tremellales	27.5	2	0	0
					
Ascomycota	Capnodiales	2.5	1	0	
	Eurotiales	0	0	2.8	0
	Glomeralles	2.5	1	0	1
	Helotiales	0	0	44.4	0
	Hypocreales	7.5	3	0	7
	Lecanorales	0	0	2.8	0
	Pleosporales	0	0	8.3	1
	Saccharomycetales	2.5	1	2.8	3
	Sordariales	2.5	1	0	1
	Xylariales	2.5	1	0	0
	Endogonales	12.5	5	0	0
Zygomycota		2.5	1	0	0
Glomermycota		2.5	1	0	0
Chytridiomycota					0

## Discussion

Few studies have examined the influence that plant species have on belowground communities within the tropics [39,40]. Moreover, most studies that have shown plant species composition negatively impacting belowground communities and ecosystem function have been from agroecosystems, grassland habitats, and invasive plant species [17-24]. Although the results from this study were based on a small sample size, they provide an example of how a naturally occurring plant can have biologically important local effects on belowground communities and ecosystem function. Although the power analyses conducted suggest a possibility for false negative (Type II errors), a number of biologically important differences were found between the soils of the bromeliad and the adjacent primary forest at *p* values from 0.07-0.13. These data suggest it is likely that we underestimate the level of difference between these two soils.

*Bromelia pinguin* is a terrestrial bromeliad found underneath forest stands throughout the Neotropics, and has been associated with inhibiting fungi in culture and impacting the belowground fungal community and associated nutrient dynamics [28,29]. The current study showed that there were biologically important differences in soil fungal community structure, rates of C and N biomass development and phenol oxidase activity between the two habitats examined, and that these differences were associated with the densities of bromeliads present. Soil moisture and pH are considered to be key drivers of belowground communities and associated ecosystem processes [41-44]. There were no observed differences in these two soil characteristics between habitats, yet there were significant differences in the density of the bromeliads between habitats. This supports the idea that this bromeliad may be driving the observed biotic and abiotic differences in these soils.

Diversity analyses showed differences in the fungal community composition and structure between bromeliad-dense and primary forest soils. The T-RFLP analyses showed that the bromeliad-dense soils had decreased diversity, richness, and community evenness within the fungal community. Results from the cloning analyses were consistent with these observations. Although a small number of clones were examined in this study, they most likely represent the more common species and are useful in gaining a preliminary profile of the fungal communities. Members of the order Helotiales consisted of 44.4% and members of the order Thelephorales consisted of 25.0% of the fungal OTUs present in primary forest soils, and were either not observed or present in very low numbers in clones from the bromeliad soils. Similarly, the most abundant fungal order found in bromeliad-dense soils, Tremellales, was not observed in primary forest soils. Representatives from the phyla Zygomycota, Glomermycota, and Chytridiomycota were also only found in the bromeliad-dense soils, with Endogonales as the only order from the Zygomycota identified. It is interesting that some Endogonalaes are known to degrade rotting wood and plant material, and others are ECM or grow along the roots of plants [45]. The absence or near absence in the bromeliad-dense soils of Helotiales and Thelephorales, which are known to be degraders of complex organic compounds, suggests that this it is possible that species within the Endogonales may be assuming a decomposition and/or ECM role that has already been filled by other groups of fungi in the primary forest soils. These taxonomic analyses showed that there was little overlap in the fungal community between these two soil types. A more detailed analysis of fungal cloning groups should be conducted to determine the degree of show differences in the composition of the decomposer community within the bromeliad soils.

Both bacteria and fungi are responsible for decomposition, yet fungi have a more integral role in the decomposition of more recalcitrant forms of C (e.g., lignin), and, therefore, shifts in fungal community structure can impact this process [46]. For example, increased diversity in of saprotrophic fungi from different orders has been linked to increased degradation of more recalcitrant forms of organic C [47]. Results from this study showed that bromeliad soils are associated with both a decrease in fungal diversity and phenol oxidase activity (an indicator of degradation of complex organic C compounds). Decreased laccase activity(one of the phenol oxidase activities) has been associated with the two most dominant fungal orders found in bromeliad soils, Tremellales and Agaricales. In a survey of 68 species of basidiomycetes testing for the presence of phenol oxidase enzymes (including laccase) involved lignin degradation, most of the species in the order Agaricales lacked all enzymes, and although laccase activity was observed in some species in the order Tremellales, activity was minimal and only observed after 21 days [48]. Others have also shown that yeasts in the order Tremellales are not involved in lignin degradation [49]. Consequently, it is possible that the observed decrease in phenol oxidase activity in bromeliad soils may be associated with the differences in fungal community present in these soils.

Metrics that project the efficiency of utilizing the organic C and N nutrients in soils can provide important indicators of a more complex soil microbial community that is more fungal-dominant [50-52]. In a previous study [29], it was determined that these bromeliad-dense soils had decreased efficiency of C use (high qCO_2_) compared to primary forest soils (low qCO_2_), suggesting that the bromeliad soil microbial community may be more directed towards respiration and maintenance, and less towards growth and C biomass development than the primary forest soil microbial community. The results from this follow-up study confirmed that the rate of use of C and N materials for biomass development was less in the bromeliad-dense than in the primary forest soils, and was associated with a decrease in fungal diversity and richness.

Decomposition of organic matter is a process that is highly sensitive to changes in community structure, and in particular, has been correlated with the number and types of soil fungal groups present [47,50]. Studies have linked differences in C and N biomass dynamics with differences in fungal community structure and plant litter composition [53]. Hanson *et al.*[54] and McGuire *et al.*[55] demonstrated resource partitioning of organic C and N substrates as fungal community composition changed. The specialization of fungal groups observed in these studies suggests that the loss of certain fungal groups and changes in fungal community structure can impact organic C and N dynamics. This is consistent with the findings in this current study in that the differences found in the fungal community between bromeliad-dense and primary forest soils were associated with changes in the rates of C and N biomass development. The correlation analyses from the current study showed negative relationships between bromeliad density, the rates of C and N biomass development and phenol oxidase activity.

## Conclusions

This study suggests that a naturally occurring plant can have biologically important effects on belowground ecosystems in established tropical forests. The two naturally occurring above- and belowground habitats studied in this current project provide future opportunities to assess the interactions between plants and microbes and how these interactions affect ecosystem function. It appears this system is exhibiting what [10] describes as top-down regulation with plant species driving the belowground community, and thus nutrient cycle dynamics.

In field studies, it is difficult to control for the fungal groups that are responsible for important ecosystem processes such as biomass development and decomposition [46]. However, the bromeliad-dense regions of these primary forest habitats provide unique natural micro-habitat experimental conditions within the primary forests that control for certain fungal groups and associated ecosystem processes. This makes these habitats useful for future studies of the fungal drivers of the C and N cycles in tropical soils. Currently, pyrosequencing analysis of sample DNA is being conducted to gain a better understanding of the influence *B. pinguin* has on the microbial community in these forests.

## Ethics statement

All research was conducted within an appropriate ethical framework. No humans, other animals or plants were involved, collected or affected, thus, no Ethics Approval was required.

## Competing interests

The authors state that there are no competing interests of any kind in relation to their work or this manuscript.

## Authors’ contributions

CL designed the project, led the collection of samples and data, analyzed the results and took the lead on writing. WDE funded the project, provided input on project design, assisted with sample collection, data analysis and interpretation, served as editor, and assisted in writing the report. Both authors read and approved the final manuscript.
